# Association of Perceived Benefit or Burden of Research Participation With Participants’ Withdrawal From Cancer Clinical Trials

**DOI:** 10.1001/jamanetworkopen.2022.44412

**Published:** 2022-11-30

**Authors:** Connie M. Ulrich, Sarah J. Ratcliffe, Qiuping Zhou, Liming Huang, Camille Hochheimer, Thomas Gordon, Kathleen Knafl, Victoria Miller, Mary D. Naylor, Marilyn M. Schapira, Therese S. Richmond, Christine Grady, Jun J. Mao

**Affiliations:** 1 Department of Biobehavioral Health Sciences, University of Pennsylvania School of Nursing, Philadelphia; 2 Public Health Sciences, University of Virginia, Charlottesville; 3 Department of Policy, Population and Systems Community, The George Washington University, Washington, DC; 4 Office of Nursing Research, University of Pennsylvania School of Nursing, Philadelphia; 5 Center for Innovative Design and Analysis, Colorado School of Public Health, Aurora; 6 Department of Psychology, University of Massachusetts Lowell, Lowell; 7 University of North Carolina at Chapel Hill, Chapel Hill; 8 Department of Pediatrics, Children’s Hospital of Philadelphia, Philadelphia, Pennsylvania; 9 Perelman School of Medicine, University of Pennsylvania, Philadelphia; 10 Center for Health Equity Research and Promotion, Philadelphia Veterans Affairs Medical Center, Philadelphia, PA; 11 Department of Bioethics, National Institutes of Health, Bethesda, Maryland; 12 Integrative Medicine, Bendheim Integrative Medicine Center, Memorial Sloan Kettering, New York, New York

## Abstract

**Importance:**

Attrition in cancer clinical trials (CCTs) can lead to systematic bias, underpowered analyses, and a loss of scientific knowledge to improve treatments. Little attention has focused on retention, especially the role of perceived benefits and burdens, after participants have experienced the trial.

**Objectives:**

To examine the association between patients’ perceived benefits and burdens of research participation and CCT retention.

**Design, Setting, and Participants:**

This survey study was conducted at a National Cancer Institute–designated comprehensive cancer center in the Northeast region of the US. The sample included adult patients with a cancer diagnosis participating in cancer therapeutic trials. Data were collected from September 2015 to June 2019. Analysis of study data was ongoing since November 2019 through October 2022.

**Exposures:**

Self-reported validated survey instrument with a list of 22 benefits and 23 burdens of research participation that can be rated by patients with a 5-point Likert scale ranging from 1 (strongly disagree) to 5 (strongly agree).

**Main Outcomes and Measures:**

A primary outcome was actual withdrawal from the CCT, and a composite outcome was composite withdrawal that included both actual withdrawal and thoughts of withdrawing. Bivariate and multivariable logistic regressions were used.

**Results:**

Among the 334 participants in the sample, the mean (SD) age was 61.9 (11.5) years and 174 women (52.1%) were included. Top-cited benefits included both aspirational and action-oriented goals, including helping others (94.2%), contributing to society (90.3%), being treated respectfully (86.2%), and hoping for a cure (86.0%). Worry over receiving a placebo (61.3%), rearranging one’s life (41.9%), and experiencing bothersome adverse effects (41.6%) were notable burdens. An increased burden score was associated with a higher probability of actual withdrawal (adjusted odds ratio [OR], 1.86; 95% CI, 1.1-3.17; *P* = .02) or composite withdrawal (adjusted OR, 3.44; 95% CI, 2.09-5.67; *P* < .001). An increased benefit score was associated with lower composite withdrawal (adjusted OR, 0.40; 95% CI, 0.24-0.66; *P* < .001). For participants who reported the benefits as being equal to or greater than the burdens, 13.4% withdrew. For those who perceived the benefits as being less than the burdens, 33.3% withdrew (adjusted OR, 3.38; 95% CI, 1.13-10.14; *P* = .03). The risk of withdrawal was even higher for the composite outcome (adjusted OR, 7.70; 95% CI, 2.76-21.48; *P* < .001).

**Conclusions and Relevance:**

This survey study found that patients perceived important benefits from CCT participation, and this perception was associated with trial retention, even among those who also perceived substantial burdens. A broader dialogue among stakeholders can inform an ethical and patient-centric focus on benefits throughout the course of a CCT to increase retention.

## Key Points

**Question:**

Is participant withdrawal from cancer clinical trials (CCTs) associated with perceived benefits or perceived burdens?

**Findings:**

In this survey study of 334 adult patients with cancer, 13.4% of those who withdrew from trial participation perceived the benefits as being equal to or greater than the burdens compared with 33.3% of patients who perceived the benefits as being less than the burdens, which was a significant difference.

**Meaning:**

Findings of this study suggest that, to increase the retention of participants in CCTs, a broader dialogue among stakeholders is needed to inform an ethical and patient-centric focus on benefits throughout the course of a CCT.

## Teaser

This survey study analyzes the association of patients' perceived benefits and burdens of research participation with retention in cancer clinical trials.

## Introduction

Participants enter clinical trials for various reasons, including altruism, hope for a cure, desire to gain time with loved ones, and to address unfinished business.^1,2,3^ For patients with cancer, a trial may be the only option for aggressive or refractory disease. Cancer is expected to take the lives of more than 605 000 people in the US in 2022.^4^ The COVID-19 pandemic has heightened treatment concerns for many patients with cancer and initially placed a strain on the research enterprise.^5,6^ Moreover, pandemic-related delays in diagnosis and treatment increased late-stage diagnoses. Participation in cancer clinical trials (CCTs) is an option for patients with cancer, albeit a challenging one given that patients may encounter difficulties in both enrollment and remaining in the trial. Issues related to recruitment, enrollment, and retention are costly for the trial and for participants who lose potential benefits.^7^ Trial withdrawal, although every participant’s right, can thwart study goals and hamper advancing novel treatments.^7^ Participant withdrawal has scientific and statistical consequences and can itself be meaningful if withdrawal suggests a lack of novel-treatment tolerance in the intervention group.

Researchers are expected to present information to interested persons to assist them in enrollment decisions. Such communication should continue throughout the trial because participants’ and researchers’ perceptions of benefits and burdens may change depending on participant response to experimental drugs, procedures, or tests and the course of illness. The Belmont Report has guided researchers on the ethical conduct of research for more than 40 years.^8^ This seminal document calls on researchers to respect human participants’ ability to make autonomous decisions and protect participants from harms while equitably distributing benefits and burdens. The Belmont Report emphasizes the systematic assessment of both risks and benefits of research participation by researchers, institutional review boards (IRBs), and prospective participants. For prospective participants, the report simply states, “the assessment will assist the determination whether or not to participate.”^8^ How individuals make this decision, what factors might alter the decision, and what elements are important for individuals to consider throughout the trial are not well known.

Little attention has focused on the weight that CCT participants give to the benefits and burdens of research participation and whether perceived benefits are a larger factor than burdens in withdrawal decisions. This study aimed to examine the association between patients’ perceived benefits and burdens of research participation and CCT retention. We were particularly interested in identifying the association of patient-reported benefit and burden scores and the difference between these scores with trial withdrawal.

## Methods

### Data and Sample

We used survey data from a mixed-methods study focused on CCT retention. The University of Pennsylvania IRB and Abramson Cancer Center approved the study. Written or oral informed consent was provided by each survey participant. We followed the Strengthening the Reporting of Observational Studies in Epidemiology (STROBE) reporting guideline.^9,10^

Patients were recruited from a National Cancer Institute–designated comprehensive cancer center in an urban Northeast region. Eligible patients participated in a CCT (phase 1, 2, or 3) and/or other clinical research treatment trial and had a cancer diagnosis (gastrointestinal, genitourinary, hematologic-lymphatic, lung, or breast or gynecological cancer). Patients were 18 years or older, spoke English or Spanish, and were able to provide informed consent. Patients were contacted by phone or at their clinic site using an IRB-approved script to obtain their oral or written informed consent. Participants were offered online, telephone, or paper-and-pencil mailed preference options to complete the survey. The web-based option was password protected to maximize data confidentiality and security. Data were collected and managed by Research Electronic Data Capture (Vanderbilt University).

Participants received $20 for survey completion. Surveys were completed between September 2015 and June 2019. A total of 498 patients consented, and 335 returned the survey (67.3%); most consented patients chose the mailed option (329 [66.0%]). The adjusted completion rate was 79% after those who died or withdrew consent before survey completion were excluded. One individual did not identify cancer type, leaving a final analytic sample of 334. The sample met the a priori power calculations with greater than 80% power to detect an odds ratio (OR) of 1.52.

### Outcomes

We examined 2 trial withdrawal outcomes: (1) primary outcome of actual withdrawal from the CCT defined by 2 variables: known trial dropout at time of survey consent and/or indication on the survey that withdrawal had already occurred, and (2) a composite outcome that included both actual withdrawal and reported thoughts of withdrawing. Participants were asked if they “ever thought of dropping out of their study,” with a response option of yes or no. If the answer was yes to either variable (already withdrew or thought of withdrawing), the composite outcome was yes. We created the composite outcome on the basis of the theory of planned behavior that intention often can indicate future behavior.^11,12,13,14^

### Primary Exposure

Using a previously developed survey instrument,^1,2^ participants rated items on a list of 22 benefits and 23 burdens of research participation on a 5-point Likert scale ranging from 1 (strongly disagree) to 5 (strongly agree). Higher scores indicated greater perceived burdens and benefits. The mean benefit and mean burden scores were calculated to yield the scale score. The instrument development study^2^ reported face and content validity and good internal consistency reliability scores for the benefit scale (0.90) and the burden scale (0.87). In this study, the internal consistency reliability scores of both scales were 0.91.

Demographic data included age; sex; race and ethnicity (self-reported by participants); marital, employment, and health insurance statuses; and educational level. Questions included the importance of patients’ spiritual-religious beliefs and previous research enrollment. Race and ethnicity were assessed to show the representativeness of the sample for generalizability reasons. Clinical data included cancer type, trial phase, functional status (measured by the Eastern Cooperative Oncology Group [ECOG] Performance Status Scale [score range: 0-5, with 0 indicating perfect health and 5 indicating death]), and presence of an advance directive.

### Statistical Analysis

Demographic data and scores were summarized overall. Mean benefit and burden scores were analyzed both as continuous and categorical variables using tertiles. The difference between the benefit and burden scores, which was calculated using mean benefit score minus mean burden score, was dichotomized to ease interpretation as follows: benefits as being greater than or equal to the burdens vs benefits as being less than the burdens. Bivariate associations between each participant characteristic (demographic and clinical) and each outcome were assessed. Characteristics with *P* < 0.2 were included in the adjusted logistic regression models as covariates. Logistic regression models examined the unadjusted associations between benefit score, burden score, and benefit-burden difference and retention outcomes. Logistic regression was repeated, adjusting for important demographic and clinical characteristics (ie, age, sex, marital and employment statuses, importance of spiritual-religious beliefs, cancer type, ECOG Performance Status score, advanced disease stage, and number of days to survey completion). We evaluated 2 multivariable models. Model 1 included the benefit and burden scores, and model 2 assessed the difference between benefits and burdens. We visually assessed how the probabilities of each outcome changed at different values of the 2 scores and the difference between them.

Missing data were less than 6% for variables in the analysis. Twenty participants (approximately 6% of the sample) had missing data for variables used in the adjusted models. We compared distributions of variables for participants with or without missing data and found no significant differences. Because the level of missing data was relatively small and there were no group differences, we used participants with complete data for regression.

Analyses were performed with SAS, version 9.4, (SAS Institute Inc). Significance was set at the α = .05 level. Analysis of study data occurred from November 2019 through October 2022.

## Results

### Characteristics

A total of 334 adult patients with cancer participated in the survey. Of these patients, 174 (52.1%) were women and 160 (47.9%) were men, with a mean (SD) age of 61.9 (11.5) years. Table 1 summarizes demographic and clinical factors and their association with withdrawal outcomes. Most patients were of White race and ethnicity (86.2%) and married or partnered (77.2%), 38.9% were working, and 41.6% were retired. More than half of patients had some or completed college education (54.8%), with 31.3% reporting postgraduate work. More than a third of the sample (37.2%) were receiving public insurance and supplemental benefits through Medicaid, Medicare, or US Department of Veterans Affairs; 48.3% had private insurance.

**Table 1.** Demographic Characteristics Overall and by Outcome

Characteristic	Overall, No. (%) (N = 334)	Withdrew (n = 47)		Withdrew or thought of withdrawing (n = 83)	
		No. (%)^a^	*P* value	No. (%)^a^	*P* value
Age, y					
Mean (SD)	61.9 (11.5)	63.7 (10.7)	.23	60.6 (11.7)	.11
Median (IQR)	64 (56-70)	65 (60-71)	NA	62 (52-69)	NA
Missing data	0	0		0	
Sex					
Male	160 (47.9)	26 (16.5)	.27	34 (21.3)	.14
Female	174 (52.1)	21 (12.2)		49 (28.2)	
Race and ethnicity (n = 333)^b^					
White	287 (86.2)	42 (14.8)	.29	70 (24.4)	.80
Other^c^	46 (13.8)	4 (8.9)		12 (26.1)	
Missing data	1	1		1	
Marital status					
Married or partnered	258 (77.2)	41 (16.1)	.08	69 (26.7)	.14
Single, widowed, or divorced	76 (22.8)	6 (8.0)		14 (18.4)	
Educational level (n = 332)					
≤High school diploma or GED certificate	46 (13.9)	6 (13.3)	.96	9 (19.6)	.82
Some college	76 (22.9)	12 (16.0)		20 (26.3)	
College degree	106 (31.9)	14 (13.3)		28 (26.4)	
Postgraduate work	104 (31.3)	15 (14.6)		26 (25.0)	
Missing data	2	0		0	
Employment status (n = 332)					
Employed	129 (38.9)	14 (10.9)	.09	37 (28.7)	.30
Retired	138 (41.6)	26 (19.4)		34 (24.6)	
Other^d^	65 (19.6)	7 (10.8)		12 (18.5)	
Missing data	2	0		0	
Importance of spiritual-religious beliefs (n = 331)					
Important	207 (62.5)	26 (12.7)	.16	46 (22.2)	.16
Somewhat important	58 (17.5)	13 (22.4)		20 (34.5)	
Not important	66 (19.9)	8 (12.5)		16 (24.2)	
Missing data	3	0		1	
Health insurance status (n = 333)					
Private	161 (48.3)	21 (13.0)	.10	46 (28.6)	.24
Public	44 (13.2)	2 (4.5)		6 (13.6)	
Public with supplemental benefits	124 (37.2)	23 (19.2)		30 (24.2)	
None	4 (1.2)	1 (25.0)		1 (25.0)	
Missing data	1	0		0	
Previous enrollment in research studies (n = 325)					
Yes	82 (25.2)	13 (15.9)	.72	24 (29.3)	.37
No	243 (74.8)	34 (14.2)		59 (24.3)	
Missing data	9	0		0	
ECOG Performance Status score					
0	184 (55.1)	22 (12.0)	.20	47 (25.5)	.75
Missing or ≥1	150 (44.9)	25 (17.0)		36 (24.0)	
Stage of disease when enrolled in current clinical trial					
I-III	148 (44.3)	13 (8.8)	.003	35 (23.6)	.08
IV	119 (35.6)	27 (23.1)		37 (31.1)	
Other	67 (20.1)	7 (10.8)		11 (16.4)	
Type of cancer					
GI or GU	117 (35.0)	18 (15.7)	.14	23 (19.7)	.03
Hematologic-lymphatic	105 (31.4)	14 (13.6)		21 (20.0)	
Lung	51 (15.3)	11 (21.6)		17 (33.3)	
Breast or gynecological	61 (18.3)	4 (6.6)		22 (36.1)	
No. of days to survey completion					
0-89	100 (29.9)	9 (9.1)	.08	19 (19.0)	.11
≥90	234 (70.1)	38 (16.5)		64 (27.4)	
With advance directive (n = 316)					
Yes	194 (61.4)	28 (14.7)	.69	47 (24.2)	.81
No	122 (38.6)	16 (13.1)		31 (25.4)	
Missing data	18	3		5	

Abbreviations: ECOG, Eastern Cooperative Oncology Group; GED, General Educational Development; GI, gastrointestinal; GU, genitourinary; NA, not applicable.

^a^ For outcomes, percentage is calculated within overall for each row and based on non-missing data.

^b^ Race and ethnicity were self-reported.

^c^ Other race and ethnicity identified were Asian (6 [1.8%]), Black (34 [10.2%]), mixed (4 [1.2%]), and other (2 [0.6%]).

^d^ Other employment status included unemployed, with disability, and homemaker.

The number of participants who withdrew from the trial was 47 (14.1%), and the number who withdrew or thought of withdrawing was 83 (24.9%). Those with stage IV disease at trial enrollment (35.6%) had a higher withdrawal rate compared with those at other disease stages (stage IV, 27 [23.1%]; stage not available, 7 [10.8%]; stages I-III, 13 [8.8%]; *P* = .003). Those with breast or gynecological cancers had higher rates of composite withdrawal than participants with other cancer types (breast or gynecological cancer, 22 [36.1%]; gastrointestinal or genitourinary cancer, 23 [19.7%]; hematologic-lymphatic cancer, 21 [20.0%]; lung cancer, 17 [33.3%]; *P* = .03). No other demographic variables were associated with withdrawal outcomes.

### Perceived Benefits and Burdens

Table 2 summarizes, both as continuous and categorical variables using tertiles, the benefit and burden scores overall and within the 2 outcomes. The mean (SD) benefit item score was 3.75 (0.61), and the mean (SD) burden item score was 2.45 (0.67). Individual survey items are listed in eTables 1 and 2 in the Supplement. Most patients wanted to help others (94.2%), even though the trial might not benefit them; wanted to contribute to society (90.3%); and help their children or other family members who might acquire the disease in the future (81.5%). Most patients (86.0%) hoped for a cure, and nearly two-thirds (63.6%) reported the benefit of access to unavailable medications and treatments. Being treated respectfully or like a person was also an important benefit (86.2%).

**Table 2.** Summary of Benefit and Burden Scores

	No. (%)			*P* value^a^	No. (%)		*P* value^a^
	Overall (n = 334)	Withdrew (n = 47)	Did not withdraw (n = 283)		Withdrew or thought of withdrawing (n = 83)	Did not withdraw nor thought of withdrawing (n = 251)	
**Benefit score**							
No.	330 (98.8)	47 (100)	279 (98.6)	.04	83 (100)	247 (98.4)	<.001
Mean (SD)	3.75 (0.61)	3.59 (0.56)	3.78 (0.62)		3.52 (0.54)	3.83 (0.62)	
Median (IQR)	3.73 (3.36 to 4.19)	3.64 (3.14 to 3.86)	3.77 (3.41 to 4.23)		3.59 (3.18 to 3.82)	3.86 (3.45 to 4.27)	
Missing data	4	0	4		0	4	
**Benefit tertiles**							
Low	111 (33.6)	17 (15.6)	92 (84.4)	.10	37 (33.3)	74 (66.7)	<.001
Medium	113 (34.2)	21 (18.6)	92 (81.4)		33 (29.2)	80 (70.8)	
High	106 (32.1)	9 (8.7)	95 (91.3)		13 (12.3)	93 (87.7)	
Missing data	4	0	4		0	4	
**Burden score**							
No.	326 (97.6)	47 (100)	275 (97.2)	.009	83 (100)	243 (96.8)	<.001
Mean (SD)	2.45 (0.67)	2.68 (0.71)	2.41 (0.66)		2.81 (0.66)	2.33 (0.63)	
Median (IQR)	2.52 (2.00 to 2.87)	2.70 (2.35 to 3.13)	2.52 (1.96 to 2.83)		2.83 (2.57 to 3.22)	2.39 (1.91 to 2.74)	
Missing data	8	0	8		0	8	
**Burden tertiles**							
Low	113 (34.7)	11 (9.8)	101 (90.2)	.17	15 (13.3)	98 (86.7)	<.001
Medium	111 (34.0)	17 (15.6)	92 (84.4)		25 (22.5)	86 (77.5)	
High	102 (31.3)	19 (18.8)	82 (81.2)		43 (42.2)	59 (57.8)	
Missing data	8	0	8		0	8	
**Benefit-burden difference**							
No.	324 (97.0)	47 (100)	273 (96.5)	.003	83 (100)	241 (96.0)	<.001
Mean (SD)	1.30 (0.94)	0.91 (1.02)	1.37 (0.91)		0.71 (0.94)	1.50 (0.85)	
Median (IQR)	1.27 (0.63 to 1.93)	0.86 (0.28 to 1.56)	1.38 (0.75 to 1.95)		0.59 (0.23 to 1.24)	1.45 (0.91 to 2.08)	
Missing data	10	0	10		0	10	

^a^ *P* values were from the χ^2^ test for categorical variables or the Wilcoxon rank sum test for continuous variables. *P* values only took into account nonmissing data.

Participants ranked their top 3 benefits: hoping for a cure (46.3%), helping future patients (32.3%), and actively treating their disease (27.5%). Worrying over receiving a placebo was the most agreed-on burden (61.3%), with some patients (29.1%) ranking it in their top 3 burdens. Other agreed-on burdens included, for example, needing to rearrange one’s life (41.9%), experiencing adverse effects (41.6%), realizing the seriousness of one’s cancer (35.4%), and paying for trial-related expenses (26.9%). Patients also ranked not deriving an individual benefit (37.1%) and experiencing adverse effects (24.5%), along with receiving a placebo, among their top 3 burdens.

### Association Between Perceived Benefit vs Burden and Withdrawal

A higher burden score was associated with a higher actual withdrawal probability (adjusted OR, 1.86; 95% CI, 1.1-3.17; *P* = .02) and composite withdrawal (adjusted OR, 3.44; 95% CI, 2.09-5.67; *P* < .001) (Table 3). Higher benefit scores were associated with a lower probability of composite withdrawal (adjusted OR, 0.40; 95% CI, 0.24-0.66; *P* < .001) but did not reach significance for actual withdrawal. Results of adjusted regression analyses are in Table 3, and the full model output is provided in eTables 3 and 4 in the Supplement. Because there were no significant interactions between benefit and burden scores, we excluded the interaction term from the models.

**Table 3.** Adjusted Logistic Regressions Assessing the Association Between Benefit and Burden Scores and the 2 Outcomes

Outcome	Adjusted^a^		Unadjusted	
	OR (95% CI)	*P* value	OR (95% CI)	*P* value
**Actual withdrawal**				
Benefit score	0.62 (0.35-1.08)	.09	0.63 (0.38-1.05)	.08
Burden score	1.86 (1.1-3.17)	.02	1.79 (1.11-2.89)	.02
**Composite withdrawal (withdrew or thought of withdrawing)**				
Benefit score	0.40 (0.24-0.66)	<.001	0.41 (0.26-0.66)	<.001
Burden score	3.44 (2.09-5.67)	<.001	3.39 (2.14-5.35)	<.001

Abbreviation: OR, odds ratio.

^a^ 10 variables were adjusted: age (continuous), sex (2 categories), marital status (2 categories), employment status (3 categories), importance of spiritual-religious beliefs (3 categories), health insurance status (4 categories), cancer type (4 categories), Eastern Cooperative Oncology Group Performance Status score (2 categories), advanced disease stage (3 categories), and number of days to survey completion (2 categories). The detailed adjusted model is provided in eTables 3 and 4 in the Supplement.

Table 4 shows the association between the benefit-burden difference score and the 2 withdrawal outcomes (full model output is provided in eTables 5 and 6 in the Supplement). Most participants (93.4%) perceived the benefits as being equal to or greater than the burdens. For those patients, 13.4% withdrew from trials compared with the 33.3% who reported the burdens as being greater than the benefits (adjusted OR, 3.38; 95% CI, 1.13-10.14). The risk of withdrawal was even higher for the composite outcome (adjusted OR, 7.70; 95% CI, 2.76-21.48; *P* < .001). Participants who perceived the burdens as being greater were 3 times more likely to withdraw (unadjusted OR, 3.24; 95% CI, 1.23-8.51). For the composite withdrawal, there was a 43.9% difference between those who perceived the benefits as being equal to or greater than the burdens (22.8%) and those who perceived the burdens as being greater than the benefits (66.7%); the latter group was nearly 7 times more likely to report composite withdrawal (unadjusted OR, 6.78; 95% CI, 2.63-17.47). These ORs were even greater in multivariable logistic regression adjusting for sociodemographic and clinical characteristics (Table 4). The greater the difference between the benefits and burdens (ie, benefit score higher than burden score), the lower the probability of each withdrawal outcome.

**Table 4.** Association Between the Benefit-Burden Difference Score and the Two Outcomes

Outcome	No. (%)			Unadjusted		Adjusted^a^	
	No	Yes	Total	OR (95% CI)	*P* value	OR (95% CI)	*P* value
**Actual withdrawal**							
Benefit ≥ burden	259 (86.6)	40 (13.4)	299 (93.4)	3.24 (1.23-8.51)	.02	3.38 (1.13-10.14)	.03
Benefit < burden	14 (66.7)	7 (33.3)	21 (6.6)				
**Composite withdrawal (withdrew or thought of withdrawing)**							
Benefit ≥ burden	234 (77.2)	69 (22.8)	303 (93.5)	6.78 (2.63-17.47)	<.001	7.70 (2.76-21.48)	<.001
Benefit < burden	7 (33.3)	14 (66.7)	21 (6.5)				

Abbreviation: OR, odds ratio.

^a^ 10 variables were adjusted: age (continuous), sex (2 categories), marital status (2 categories), employment status (3 categories), importance of spiritual-religious beliefs (3 categories), health insurance status (4 categories), cancer type (4 categories), Eastern Cooperative Oncology Group Performance Status score (2 categories), advanced disease stage (3 categories), and number of days to survey completion (2 categories). The detailed adjusted model is provided in eTables 5 and 6 in the Supplement.

### Estimated Probabilities

Figure, A shows probabilities of actual withdrawal at different benefit and burden scores and difference scores. The probability was low when the benefit score was high or when it was much higher than the burden score. The probability increased as the burden score increased. Figure, B shows probabilities of the composite withdrawal at different score values, with patterns similar to those shown in Figure, A. Specifically, as the benefit-burden difference became greater (ie, when the benefit score was higher than the burden score), patients had a significantly lower probability of each outcome.

**Figure.**
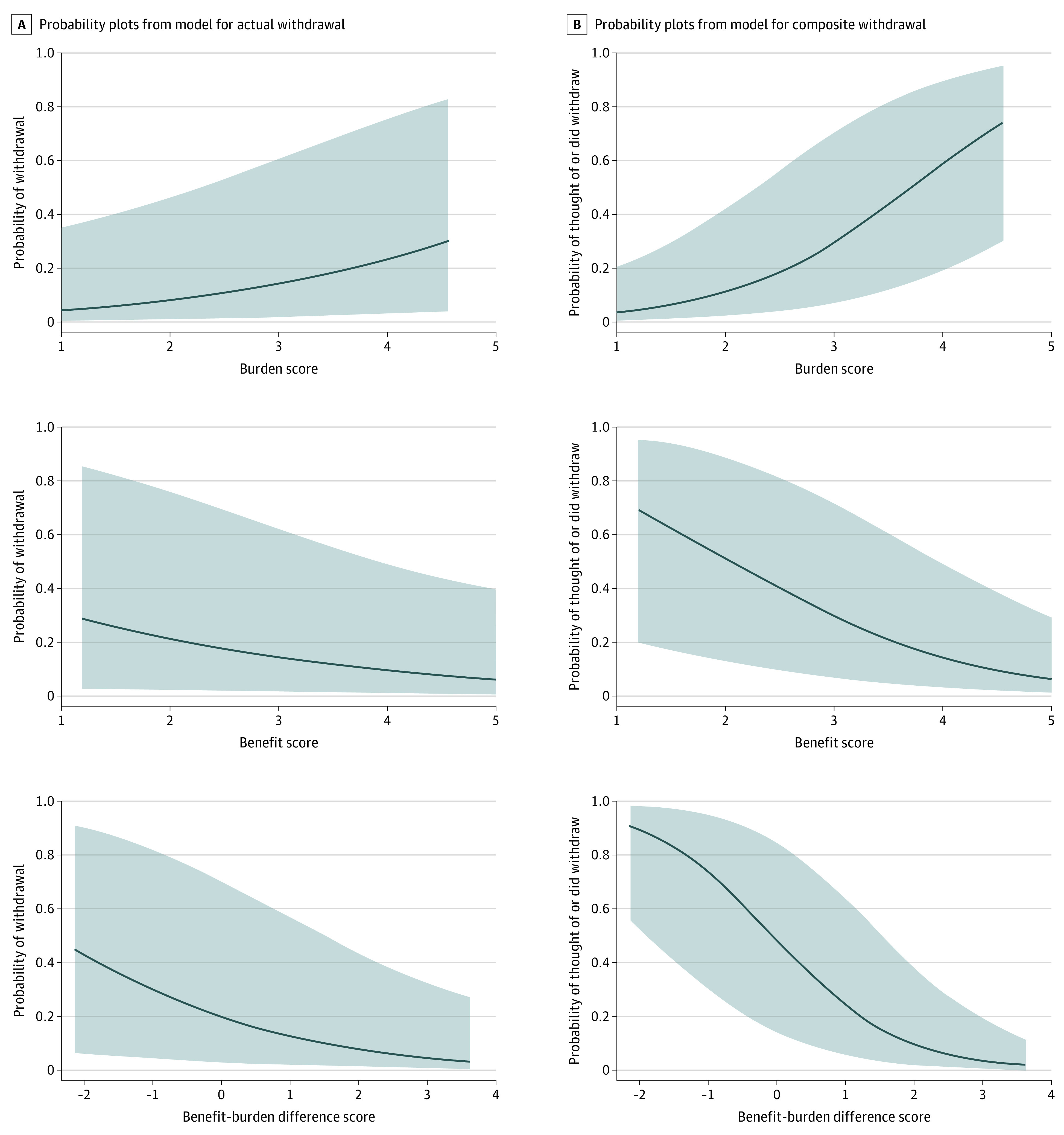
Estimated Probabilities and 95% CIs of Each Outcome by Changes in the Benefit and Burden Scores and Changes in the Benefit-Burden Difference A, The graphs show how the probability of withdrawal changed with the benefit score, burden score, and the difference between the benefit and burden scores. The probability of withdrawal increased with the burden score and decreased with the benefit score and the benefit-burden difference. B, The graphs show the probability of thought of withdrawing or actually withdrawing. There was a sharp decline in the probability of thought of withdrawing or actually withdrawing as the benefit score became greater than the burden score. The shading indicates the 95% CIs.

## Discussion

Risk-benefit assessments are essential in human participant protection. The general assumption is that perceived burdens of research participation and exposure to experimental drugs, tests, and procedures weigh heavily in enrollment and withdrawal decisions.^1^ Findings of the present study confirmed this hypothesis. Those who perceived more burden than benefit were more likely to withdraw or think about withdrawing. Similarly, higher perceived benefits reduced the likelihood of composite withdrawal. The study showed a complex interplay between perceived burdens and benefits. When perceived benefits were equal to or greater than perceived burdens, most participants were less likely to withdraw than those who perceived the burdens to be greater than the benefits. How participants think about benefits and burdens in a research trial may be different from how researchers and IRBs discern the trial’s acceptability.

First, findings suggested that research participation burdens remain a salient concern, and patients withdraw because of these concerns; trial and cancer-related burdens are also consequential. More than 40% of patients experienced bothersome adverse effects from trial medications (41.6%) and had to rearrange their lives to participate (41.9%); 61.3% worried about receiving a placebo, 32.5%% worried that the trial would not benefit them, and 26.9% reported paying for trial-related expenses out of pocket. The use of telemedicine for clinical care or clinical trial visits, whose increase was spurred on by the COVID-19 pandemic, can reduce time and financial burden for patients; future research is needed on the use of telemedicine to monitor toxic effects and follow-up needs.^15^ The experimental medicines used in CCTs can create life-restricting fatigue, sickness or retching, and other adverse effects.^16^ Moreover, cancer itself can cause neuropathies, pain, and psychological distress, among other events.^16,17^ However, we found that perceived benefits of research participation were important to all patients and were a factor in trial retention, even in the face of many perceived burdens.

Second, the general focus on risks far exceeds the focus on benefits of research participation; the current study findings regarding the importance of perceived benefits may be useful in addressing this imbalance. In many ways, the historical and regulatory focus on risk provides the foundation for protecting human participants and avoiding potential harms. Conversely, potential research benefits are often underplayed by researchers but are clearly important to patients’ CCT enrollment and retention. As this and other studies have reported, the potential benefit of hoping for a cure or slowing disease progression is a powerful motivator for enrollment.^18^ Hope of remission, cure, or improvement in life quality is welcome for any patient with cancer, and 35.6% of the sample had stage IV cancer at enrollment. Should hope for a cure, however, be considered as an important benefit of participation? As several authors have suggested, hope and optimism can be factors in good outcomes, but they can also foster unrealistic expectations.^19,20,21^

We need to better understand the different ways that patients, IRBs, and investigators may view the benefits of research participation. Perceptions of the benefits and burdens may vary depending on the trial phase and designated aims. Phase 1 trials, for example, test the safety of an experimental drug, and although patients may benefit (eg, remission and higher quality of life), the goal of such trials is not to cure disease. On the other hand, phase 3 trials test whether a new and previously tested drug is better than the standard of care. Further work is needed to examine how participants’ perceptions may vary across trial phases and designs (eg, inclusion of placebo control, adaptive trial design, and platform-based design). Moreover, early and ongoing discussions of expectations about benefits may not only help with the trial enrollment decision but may also foster trial retention. There may be a disconnect between patients’ expectations of the benefits of participation and their actual experience. This disconnect may be a factor in self-withdrawal or thoughts about withdrawal. More than one-third of the sample (37.1%) reported fear that the trial might not be beneficial for them as a top-ranked burden.

Third, the results highlighted the importance of understanding how trial participants weigh the benefits and burdens in withdrawal decisions; fewer participants withdrew who rated the benefits higher than the burdens. Investigators rely on IRBs to identify the benefit-burden balance of research protocols. However, Churchill et al^22^ found a lack of uniformity in how IRBs assessed research benefits: some recognized both societal and participant benefits, whereas others recognized one or the other. The authors further attempted to categorize benefits according to the sentinel work of King^23^ as direct benefits from the intervention and collateral benefits. However, there is much diversity in how these assessments are considered.

Although reporting altruistic reasons (eg, giving back to society) for research participation could reflect a social desirability bias, the data from this study suggested that altruism also may be a valid reason. Future research should examine whether altruistic giving by research participants is associated with adverse outcomes or motivates a sense of solidarity, community, and pride.

When ranking their top 3 benefits, patients listed items that reflected both aspirational and action-oriented goals (ie, hoping for a cure, helping future patients, and actively treating their disease). These benefits might not necessarily fit into the 2 defined-benefit categories of King.^23^ For example, hoping for a cure might be perceived as a direct benefit because of the potential nature of the experimental intervention, but it could also be a collateral benefit of simply participating and giving something back or the psychological benefit of trying.

How are we to understand hope as a benefit in the context of clinical trials? Gwinn and Hellman^24^^(p9)^ stated that “hope is the belief that your future can be brighter and better than your past and that you actually have a role to play in making it better.” Trials provide a pathway for individuals to actively engage in their treatment and to hope. More than 15 years ago, Churchill et al^22^ wrote that understanding the nature, likelihood, and magnitude of benefits in research was underdeveloped. Since then, we have made limited progress, especially in understanding patients’ perceptions. Patients may perceive research benefits as physical, psychological, educational, economic, or familial and as meeting their short-, intermediate-, or long-term care goals.^1^

These findings raise important questions for researchers, ethicists, and IRBs on the details of informed consent. Informed consent documents are replete with information about potential risks, but most benefit statements are restrained. Researchers are often careful to state that there is no direct benefit to participation but that participation might contribute to knowledge and help future patients. As noted in this study, however, patients might feel that it is a benefit to participate in something that can help others, including their children who might later acquire the disease. This *epidemiology of altruism*^25^ of helping future others is usually a single statement in the informed consent document, but how it can be categorized within the benefit domain remains elusive. There are many perceived benefits to CCT participation (eg, feeling more control over one’s diagnosis or being treated in a respectful and equitable manner), and a broader dialogue among all relevant stakeholders could guide the ethical and patient-centric emphasis on these benefits.

### Strengths and Limitations

This study had numerous strengths. First, it included a large sample of CCT participants with varying tumor types. Second, it used a validated instrument to enable participant assessment of benefits and burdens of trial participation.

This study also had several limitations. First, it was conducted at a single institution. Patients at other institutions may hold different views of CCT participation as well as perceived benefits and burdens. Second, the sample comprised highly educated and mostly White individuals. We do not know whether the perceived benefits would still outweigh the perceived burdens in decision-making about trial withdrawal for populations in other educational level and racial and ethnic groups. Work is needed on differences due to social determinants of health, including culture, social position, and access to clinical trials. Third, this cross-sectional survey study captured views at one point in time in patients’ research participation. Whether and when benefit perceptions change is not known, and they may change at different times for each individual and type and phase of trials. Although we found no temporal differences in participants’ rates of withdrawal or perceptions, recall bias was a potential limitation. We did not specify the kinds of benefits or burdens that may be associated with greater withdrawal from CCTs; future research may help clarify what is important to patients and families and why some patients remain in CCTs even when the perceived burdens are greater than the benefits. Fourth, the study captured self-reported responses to complex phenomena, and although we attempted to evaluate the retention status of participants as much as possible, there is always the potential for underreporting or overreporting depending on available information. However, self-reporting was also a strength because the average participant was in the best position to describe what they wanted to know before enrollment or what has happened during trial participation.^26^ Patients’ voices help ensure representation and informed decisions that support research ethics guidelines in a just fashion.

## Conclusions

The findings of how perceptions of benefits and burdens were associated with CCT withdrawal outcomes provide novel and foundational evidence of the importance of understanding these perceptions for trial retention. Thus, IRBs, researchers, ethicists, and patients and their families should discuss trial benefits and burdens in a reasonable way without being perceived as unduly powerful. Such discussions may vary across phases and types of trial. Meaningful change requires input from participants given that they experience the ups and downs of trial participation and can provide first-hand information about their experiences.^26^ Protection of human participants is critical, but more research is needed on how participants perceive benefits, the different types and categories of benefits, and implications of perceived benefits for retention to elucidate the role of benefits compared with the risks and burdens that participants are asked to bear. Progress in research ethics occurs only through the rigorous examination of its processes; it is past time to understand the range of what is beneficial in research and to revisit regulatory guidelines that address this concern.

## Notes

Supplement. **eTable 1.** Benefit Items **eTable 2.** Burden Items **eTable 3.** Logistic Regressions Showing the Adjusted Associations Between Benefit/Burden Scores and Withdrawal (N for Analysis=314) **eTable 4.** Logistic Regressions Showing the Adjusted Associations Between Benefit/Burden Scores and Thoughts of Withdrawing or Did Withdraw (N for Analysis=318) **eTable 5.** Logistic Regressions Showing the Adjusted Associations Between Benefit/Burden Difference Scores and Withdrawal (N for Analysis=314) **eTable 6.** Logistic Regressions Showing the Adjusted Associations Between Benefit/Burden Difference Scores and Thoughts of Withdrawing or Did Withdraw (N for Analysis=318)

## References

[1] Ulrich CM, Knafl KA, Ratcliffe SJ, . Developing a model of the benefits and burdens of research participation in cancer clinical trials. AJOB Prim Res. 2012;3(2):10-23. doi:10.1080/21507716.2011.653472 24748992 PMC3989990

[2] Ulrich CM, Zhou QP, Ratcliffe SJ, . Development and preliminary testing of the perceived benefit and burden scales for cancer clinical trial participation. J Empir Res Hum Res Ethics. 2018;13(3):230-238. doi:10.1177/1556264618764730 29631487 PMC6091872

[3] Castillo AG, Jandorf L, Thélémaque LD, King S, Duhamel K. Reported benefits of participation in a research study. J Community Health. 2012;37(1):59-64. doi:10.1007/s10900-011-9416-0 21644025 PMC4399714

[4] Siegel RL, Miller KD, Fuchs HE, Jemal A. Cancer statistics, 2022. CA Cancer J Clin. 2022;72(1):7-33. doi:10.3322/caac.21708 35020204

[5] Patt D, Gordan L, Diaz M, . Impact of COVID-19 on cancer care: how the pandemic is delaying cancer diagnosis and treatment for American seniors. JCO Clin Cancer Inform. 2020;4:1059-1071. doi:10.1200/CCI.20.00134 33253013 PMC7713534

[6] Broom A, Kenny K, Page A, . The paradoxical effects of COVID-19 on cancer care: current context and potential lasting impacts. Clin Cancer Res. 2020;26(22):5809-5813. doi:10.1158/1078-0432.CCR-20-2989 32816894

[7] Fogel DB. Factors associated with clinical trials that fail and opportunities for improving the likelihood of success: a review. Contemp Clin Trials Commun. 2018;11:156-164. doi:10.1016/j.conctc.2018.08.001 30112460 PMC6092479

[8] National Commission for the Protection of Human Subjects of Biomedical and Behavioral Research. The Belmont Report: ethical principles and guidelines for the protection of human subjects of research. 1978. Accessed October 28, 2022. https://www.hhs.gov/ohrp/sites/default/files/the-belmont-report-508c_FINAL.pdf 25951677

[9] von Elm E, Altman DG, Egger M, Pocock SJ, Gøtzsche PC, Vandenbroucke JP; STROBE Initiative. Strengthening the Reporting of Observational Studies in Epidemiology (STROBE) statement: guidelines for reporting observational studies. BMJ. 2007;335(7624):806-808. doi:10.1136/bmj.39335.541782.AD 17947786 PMC2034723

[10] Cuschieri S. The STROBE guidelines. Saudi J Anaesth. 2019;13(suppl 1):S31-S34. doi:10.4103/sja.SJA_543_18 30930717 PMC6398292

[11] Ajzen I. The theory of planned behavior: frequently asked questions. Hum Behav Emerg Technol. 2020;2 (4):314-324. doi:10.1002/hbe2.195

[12] Ajzen I. The theory of planned behavior. Organ Behav Hum Decis Process. 1991;50(2):179-211. doi:10.1016/0749-5978(91)90020-T

[13] Sheeran P. Intention-behavior relations: a conceptual and empirical review. Eur Rev Soc Psychol. 2002;12(1):1-36. doi:10.1080/14792772143000003

[14] Sheeran P, Webb TL. The intention-behavior gap. Soc Personal Psychol Compass. 2016;10(9):503-518. doi:10.1111/spc3.12265

[15] Li BT, Daly B, Gospodarowicz M, . Reimagining patient-centric cancer clinical trials: a multi-stakeholder international coalition. Nat Med. 2022;28(4):620-626. doi:10.1038/s41591-022-01775-6 35440725

[16] Bouchard LC, Aaronson N, Gondek K, Cella D. Cancer symptom response as an oncology clinical trial end point. Expert Rev Qual Life Cancer Care. 2018;3(2-3):35-46. doi:10.1080/23809000.2018.1483193 31020045 PMC6476191

[17] Cleeland CS, Zhao F, Chang VT, . The symptom burden of cancer: evidence for a core set of cancer-related and treatment-related symptoms from the Eastern Cooperative Oncology Group Symptom Outcomes and Practice Patterns Study. Cancer. 2013;119(24):4333-4340. doi:10.1002/cncr.28376 24114037 PMC3860266

[18] Todd AM, Laird BJ, Boyle D, Boyd AC, Colvin LA, Fallon MT. A systematic review examining the literature on attitudes of patients with advanced cancer toward research. J Pain Symptom Manage. 2009;37(6):1078-1085. doi:10.1016/j.jpainsymman.2008.07.009 19419837

[19] Jansen LA. The problem with optimism in clinical trials. IRB. 2006;28(4):13-19. 17036434

[20] Jansen LA. The optimistic bias and illusions of control in clinical research. IRB. 2016;38(2):8-14. 27188031

[21] Laranjeira C, Querido A. Hope and optimism as an opportunity to improve the “positive mental health” demand. Front Psychol. 2022;13:827320. doi:10.3389/fpsyg.2022.827320 35282230 PMC8907849

[22] Churchill LR, Nelson DK, Henderson GE, . Assessing benefits in clinical research: why diversity in benefit assessment can be risky. IRB. 2003;25(3):1-8. doi:10.2307/3564297 14569987

[23] King NM. Defining and describing benefit appropriately in clinical trials. J Law Med Ethics. 2000;28(4):332-343. doi:10.1111/j.1748-720X.2000.tb00685.x 11317426

[24] Gwinn C, Hellman C. Hope Rising: How the Science of Hope Can Change Your Life. Morgan James Publishing; 2022.

[25] Nowak MA, Roch S. Upstream reciprocity and the evolution of gratitude. Proc Biol Sci. 2007;274(1610):605-609. doi:10.1098/rspb.2006.0125 17254983 PMC2197219

[26] Dresser R. Silent Partners: Human Subjects and Research Ethics. Oxford University Press; 2017.

