# Supplementary material for: Association of Perceived Benefit or Burden of Research Participation With Participants’ Withdrawal From Cancer Clinical Trials
Source: JAMA Netw Open. 2022 Nov 30;5(11):e2244412. doi: 10.1001/jamanetworkopen.2022.44412 (PMC9713607; doi:10.1001/jamanetworkopen.2022.44412)
Supplement: Supplement. — eTable 1. Benefit Items eTable 2. Burden Items eTable 3. Logistic Regressions Showing the Adjusted Associations Between Benefit/Burden Scores and Withdrawal (N for Analysis=314) eTable 4. Logistic Regressions Showing the Adjusted Associations Between Benefit/Burden Scores and Thoughts of Withdrawing or Did Withdraw (N for Analysis=318) eTable 5. Logistic Regressions Showing the Adjusted Associations Between Benefit/Burden Difference Scores and Withdrawal (N for Analysis=314) eTable 6. Logistic Regressions Showing the Adjusted Associations Between Benefit/Burden Difference Scores and Thoughts of Withdrawing or Did Withdraw (N for Analysis=318) [file jamanetwopen-e2244412-s001.pdf]

Supplemental Online Content

Ulrich CM, Ratcliffe SJ, Zhou Q, et al. Association of perceived benefit or burden of research participation with participants' withdrawal from cancer clinical trials. *JAMA Netw Open*. 2022;5(11):e2244412. doi:10.1001/jamanetworkopen.2022.44412

eTable 1. Benefit Items

eTable 2. Burden Items

eTable 3. Logistic Regressions Showing the Adjusted Associations Between Benefit/Burden Scores and Withdrawal (N for Analysis=314)

eTable 4. Logistic Regressions Showing the Adjusted Associations Between Benefit/Burden Scores and Thoughts of Withdrawing or Did Withdraw (N for Analysis=318)

eTable 5. Logistic Regressions Showing the Adjusted Associations Between Benefit/Burden Difference Scores and Withdrawal (N for Analysis=314)

eTable 6. Logistic Regressions Showing the Adjusted Associations Between Benefit/Burden Difference Scores and Thoughts of Withdrawing or Did Withdraw (N for Analysis=318)

This supplemental material has been provided by the authors to give readers additional information about their work.

© 2022 Ulrich CM et al. *JAMA Network Open.*

eTable 1. Benefit Items

Benefit	% Agree or strongly agree, N (%)	% in top 3 benefits[1], N (%)
1. I am providing a valuable contribution to society	298 (90.3)	78 (24.9)
2. I might help future patients with my disease (even though my participation might not help me)	311 (94.2)	101 (32.3)
3. My disease is watched more closely than it would otherwise be	232 (70.5)	55 (17.6)
4. I am treated like a person and not a number by my research team	282 (86.2)	19 (6.1)
5. I worry less about my disease when I participate in research	153 (46.6)	6 (1.9)
6. It lessens my stress associated with my disease	154 (47.1)	3 (1.0)
7. It allows me to have some control over my disease	180 (55.0)	16 (5.1)
8. It may help my children or other family members in the future	268 (81.5)	61 (19.5)
9. It gives me information about my disease that I would not otherwise have	202 (61.8)	17 (5.4)
10. I feel more informed about my specific type of cancer and the treatments	218 (66.1)	11 (3.5)
11. It helps me know what to expect about my specific type of cancer	187 (56.8)	6 (1.9)
12. I am able to extend my life	212 (64.6)	64 (20.4)
13. It gives me a sense of hope about my disease	262 (79.6)	63 (20.1)
14. I am hoping for a cure	282 (86.0)	145 (46.3)
15. It helps to pay for the costs of drugs and other medications or tests that I might not otherwise be able to afford	117 (35.5)	19 (6.1)
16. My insurance will likely cover all of my research expenses	129 (39.7)	5 (1.6)
17. It is a way for me to actively treat the disease	241 (73.9)	86 (27.5)
18. It does not interfere with my other life responsibilities	202 (61.4)	24 (7.7)
19. It may reduce my risk of cancer in the future	181 (55.0)	56 (17.9)
20. I trust my researcher knows what is best for me	256 (77.6)	30 (9.6)
21. I receive money for participating	31 (9.5)	2 (0.6)
22. I have access to drugs and other medicines/test that are not available to me otherwise	208 (63.6)	68 (21.7)

[1] 21 patients did not list any top benefits.

© 2022 Ulrich CM et al. *JAMA Network Open.*

eTable 2. Burden Items

Burden	% Agree or strongly agree, N (%)	% in top 3 burdens[1], N (%)
1. I have additional personal responsibilities that I did not expect	64 (19.5)	14 (5.0)
2. It has added stress to managing my cancer (for example, trying to coordinate services for my care in seeing different doctors, bills, paperwork, traveling with X-rays and MRIs etc.)	75 (22.8)	32 (11.5)
3. I have had to rearrange my life in order to take part in research (for example, travel time, being out of work, meeting scheduled appointments)	138 (41.9)	67 (24.1)
4. It has made me realize the seriousness of my cancer	116 (35.4)	37 (13.3)
5. There are unknown side effects that are potentially life threatening	97 (29.8)	40 (14.4)
6. I have experienced bothersome side effects	136 (41.6)	68 (24.5)
7. My quality of life is less	58 (18.6)	21 (7.6)
8. I find it difficult to balance my family needs with my own needs for treatment	37 (11.4)	14 (5.0)
9. I worry that it is difficult for my family (including children) to see me take part in a research study for my cancer	42 (12.8)	20 (7.2)
10. I am not learning anything more about my cancer from being in a research study	51 (15.6)	25 (9.0)
11. It might not benefit me	106 (32.5)	103 (37.1)
12. I would be very disappointed if I received a placebo (an inactive substance) instead of the treatment	193 (61.3)	81 (29.1)
13. It makes me worry about other family members who could be at risk for cancer	106 (33.0)	47 (16.9)
14. The amount of information that I needed to understand (to be in the study) is overwhelming	35 (10.8)	7 (2.5)
15. I worry that I did not understand everything about the research and what it meant when I agreed to be in the study	24 (7.4)	13 (4.7)
16. I have to rely on others for my needs (financial, personal care, support)	54 (16.7)	28 (10.1)
17. My insurance does not cover all the costs of being in the study	68 (21.2)	24 (8.6)
18. Others perceive me as a guinea pig	18 (5.6)	2 (0.7)
19. I sometimes feel like a guinea pig	42 (13.0)	11 (4.0)
20. I am uncertain if the research is helping or hurting me	73 (22.5)	61 (21.9)
21. I often wonder if the researcher is not telling me everything about my treatment	24 (7.3)	9 (3.2)
22. I am tired because of my research participation	64 (19.8)	27 (9.7)
23. It is costing me money out of pocket	86 (26.9)	38 (13.7)

[1] 56 patients did not list any top burdens.

© 2022 Ulrich CM et al. *JAMA Network Open.*

eTable 3. Logistic Regressions Showing the Adjusted Associations Between Benefit/Burden Scores and Withdrawal (N for Analysis=314)

variable	category	odds ratio (95% CI)	p value
Benefit score		0.62 (0.35, 1.08)	0.09
Burden score		1.86 (1.1, 3.17)	0.02
Age		1 (0.95, 1.05)	0.95
Sex	Female	ref	
Sex	Male	0.82 (0.38, 1.79)	0.62
Marital status	Married/partnered	ref	
Marital status	Single/widowed/divorced	0.45 (0.16, 1.28)	0.14
Employment status	Employed	ref	
Employment status	Retired	1.94 (0.71, 5.31)	0.20
Employment status	Other	0.87 (0.29, 2.56)	0.80
Importance of spiritual beliefs	Important	ref	
Importance of spiritual beliefs	Somewhat important	1.36 (0.57, 3.22)	0.49
Importance of spiritual beliefs	Not important	1.07 (0.42, 2.76)	0.89
Health insurance	Private	ref	
Health insurance	Public	0.19 (0.04, 1.02)	0.05
Health insurance	Public with supplemental	0.96 (0.37, 2.51)	0.93
Health insurance	None	2.78 (0.22, 35.3)	0.43
Performance status (ECOG)	Zero	ref	
Performance status (ECOG)	Non-zero	1.1 (0.52, 2.34)	0.80
Days to survey completion	0-89 days	ref	
Days to survey completion	90+ days	1.89 (0.8, 4.48)	0.15
Type of cancer	GI/GU	ref	
Type of cancer	Hematologic	1.54 (0.51, 4.63)	0.45
Type of cancer	Lung	1.03 (0.39, 2.73)	0.96
Type of cancer	Breast/GYN	0.4 (0.11, 1.46)	0.16
Stage of disease when enrolled in current clinical	Stages 1 to 3	ref	
Stage of disease when enrolled in current clinical	IV	3 (1.28, 7.01)	0.01
Stage of disease when enrolled in current clinical	Other	0.69 (0.19, 2.47)	0.57

© 2022 Ulrich CM et al. *JAMA Network Open.*

eTable 4. Logistic Regressions Showing the Adjusted Associations Between Benefit/Burden Scores and Thoughts of Withdrawing or Did Withdraw (N for Analysis=318)

variable	category	odds ratio (95% CI)	p value
Benefit score		0.4 (0.24, 0.66)	< 0.001
Burden score		3.44 (2.09, 5.67)	< 0.001
Age		1 (0.96, 1.03)	0.87
Sex	Female	ref	
Sex	Male	0.66 (0.33, 1.31)	0.23
Marital status	Married/partnered	ref	
Marital status	Single/widowed/divorced	0.63 (0.29, 1.39)	0.25
Employment status	Employed	ref	
Employment status	Retired	0.81 (0.35, 1.86)	0.62
Employment status	Other	0.44 (0.18, 1.05)	0.07
Importance of spiritual beliefs	Important	ref	
Importance of spiritual beliefs	Somewhat important	1.53 (0.71, 3.3)	0.28
Importance of spiritual beliefs	Not important	1.12 (0.52, 2.41)	0.76
Health insurance	Private	ref	
Health insurance	Public	0.41 (0.13, 1.25)	0.12
Health insurance	Public with supplemental	0.83 (0.36, 1.92)	0.66
Health insurance	None	0.69 (0.05, 8.9)	0.78
Performance status (ECOG)	Zero	ref	
Performance status (ECOG)	Non-zero	0.99 (0.51, 1.92)	0.98
Days to survey completion	0-89 days	ref	
Days to survey completion	90+ days	1.37 (0.69, 2.71)	0.37
Type of cancer	GI/GU	ref	
Type of cancer	Hematologic	1.71 (0.62, 4.67)	0.30
Type of cancer	Lung	2.42 (1, 5.88)	0.05
Type of cancer	Breast/GYN	2.02 (0.79, 5.17)	0.14
Stage of disease when enrolled in current clinical	Stages 1 to 3	ref	
Stage of disease when enrolled in current clinical	IV	2.16 (1.06, 4.4)	0.03
Stage of disease when enrolled in current clinical	Other	0.81 (0.26, 2.49)	0.71

© 2022 Ulrich CM et al. *JAMA Network Open.*

eTable 5. Logistic Regressions Showing the Adjusted Associations Between Benefit/Burden Difference Scores and Withdrawal (N for Analysis=314)

variable	category	odds ratio 95% CI	p value
Benefit-burden difference	benefit >= burden	ref	
Benefit-burden difference	benefit < burden	3.38 (1.13, 10.14)	0.03
Age		1 (0.95, 1.04)	0.84
Sex	Female	ref	
Sex	Male	0.76 (0.35, 1.63)	0.48
Marital status	Married/partnered	ref	
Marital status	Single/widowed/divorced	0.42 (0.15, 1.18)	0.1
Employment status	Employed	ref	
Employment status	Retired	1.88 (0.7, 5.09)	0.21
Employment status	Other	0.87 (0.3, 2.54)	0.79
Importance of spiritual beliefs	Important	ref	
Importance of spiritual beliefs	Somewhat important	1.65 (0.72, 3.8)	0.24
Importance of spiritual beliefs	Not important	1.12 (0.44, 2.87)	0.81
Health insurance	Private	ref	
Health insurance	Public	0.2 (0.04, 1.06)	0.06
Health insurance	Public with supplemental	0.98 (0.39, 2.45)	0.96
Health insurance	None	2.47 (0.21, 29.04)	0.47
Performance status (ECOG)	Zero	ref	
Performance status (ECOG)	Non-zero	1.13 (0.54, 2.37)	0.74
Days to survey completion	0-89 days	ref	
Days to survey completion	90+ days	1.78 (0.76, 4.19)	0.19
Type of cancer	GI/GU	ref	
Type of cancer	Hematologic	1.55 (0.53, 4.57)	0.42
Type of cancer	Lung	1.07 (0.41, 2.8)	0.9
Type of cancer	Breast/GYN	0.38 (0.1, 1.39)	0.14
Stage of disease when enrolled in current clinical	Stages 1 to 3	ref	
Stage of disease when enrolled in current clinical	IV	2.85 (1.24, 6.58)	0.01
Stage of disease when enrolled in current clinical	Other	0.69 (0.2, 2.36)	0.55

© 2022 Ulrich CM et al. *JAMA Network Open.*

eTable 6. Logistic Regressions Showing the Adjusted Associations Between Benefit/Burden Difference Scores and Thoughts of Withdrawing or Did Withdraw (N for Analysis=318)

variable	category	odds ratio (95% CI)	p value
Benefit-burden difference	benefit >= burden	ref	
Benefit-burden difference	benefit < burden	7.7 (2.76, 21.48)	< 0.001
Age		0.99 (0.96, 1.03)	0.76
Sex	Female	ref	
Sex	Male	0.58 (0.3, 1.13)	0.11
Marital status	Married/partnered	ref	
Marital status	Single/widowed/divorced	0.56 (0.27, 1.17)	0.12
Employment status	Employed	ref	
Employment status	Retired	0.85 (0.39, 1.87)	0.69
Employment status	Other	0.49 (0.21, 1.14)	0.1
Importance of spiritual beliefs	Important	ref	
Importance of spiritual beliefs	Somewhat important	1.93 (0.95, 3.93)	0.07
Importance of spiritual beliefs	Not important	1.26 (0.61, 2.61)	0.53
Health insurance	Private	ref	
Health insurance	Public	0.44 (0.15, 1.3)	0.14
Health insurance	Public with supplemental	0.81 (0.37, 1.75)	0.59
Health insurance	None	0.63 (0.05, 7.7))	0.72
Performance status (ECOG)	Zero	ref	
Performance status (ECOG)	Non-zero	1.05 (0.57, 1.96)	0.87
Days to survey completion	0-89 days	ref	
Days to survey completion	90+ days	1.16 (0.61, 2.22)	0.65
Type of cancer	GI/GU	ref	
Type of cancer	Hematologic	1.72 (0.68, 4.35)	0.26
Type of cancer	Lung	2.31 (1.01, 5.29)	0.05
Type of cancer	Breast/GYN	1.81 (0.75, 4.35)	0.19
Stage of disease when enrolled in current clinical	Stages 1 to 3	ref	
Stage of disease when enrolled in current clinical	IV	2.03 (1.04, 3.98)	0.04
Stage of disease when enrolled in current clinical	Other	0.77 (0.27, 2.18)	0.63

© 2022 Ulrich CM et al. *JAMA Network Open.*
